# Safety of pazopanib and sunitinib in treatment-naive patients with metastatic renal cell carcinoma: Asian versus non-Asian subgroup analysis of the COMPARZ trial

**DOI:** 10.1186/s13045-018-0617-1

**Published:** 2018-05-22

**Authors:** Jun Guo, Jie Jin, Mototsugu Oya, Hirotsugu Uemura, Shunji Takahashi, Katsunori Tatsugami, Sun Young Rha, Jae-Lyun Lee, Jinsoo Chung, Ho Yeong Lim, Hsi Chin Wu, Yen Hwa Chang, Arun Azad, Ian D. Davis, Marlene J. Carrasco-Alfonso, Bhupinder Nanua, Jackie Han, Qasim Ahmad, Robert Motzer

**Affiliations:** 1 Department Urology and Melanoma, Peking University Cancer Hospital and Institute, 52 Fucheng Road, Hai-dian District, Beijing, 100142 China; 2 Department of Urology, Peking University First Hospital, Beijing, China; 3 Department of Urology, Keio University School of Medicine, Tokyo, Japan; 4 Department of Urology, Kindai University Faculty of Medicine, 377-2 Ohnohigashi, Osakasayama, Osaka 589-8511 Japan; 5 Department of Medical Oncology, Cancer Institute Hospital, Japanese Foundation for Cancer Research, Tokyo, 135-8550 Japan; 6 Department of Urology, Graduate School of Medical Sciences, Kyushu University, Fukuoka, 812-8582 Japan; 7 Yonsei Cancer Center, Yonsei University College of Medicine, Seoul, South Korea; 8 Department of Oncology and Internal Medicine, Asan Medical Center, University of Ulsan College of Medicine, Seoul, 138-736 South Korea; 9 Department of Urology, Center for Prostate Cancer, National Cancer Center, 323 Ilsan-ro, Ilsandong-gu, Goyang-si, Gyeonggi-do 10408 South Korea; 10 Division of Hematology-Oncology, Samsung Medical Center, Sungkyunkwan University, Seoul, Korea; 11 Department of Urology, China Medical University Hospital, Taichung, Taiwan; 12 Division of Urology, Department of Surgery, Taipei Veterans General Hospital, Taipei, Taiwan; 13 Department of Medicine, Monash University, 246 Clayton Road, Melbourne, VIC 3168 Australia; 14 Monash University, Eastern Health Clinical School, Level 2, 5 Arnold St, Box Hill, VIC 3128 Australia; 15 Novartis Pharmaceuticals Corporation, East Hanover, NJ USA; 16 Novartis Pharmaceuticals Corporation, Basel, Switzerland; 17 Department of Medicine, Genitourinary Oncology Service, Memorial Sloan-Kettering Cancer Center, 1275 York Avenue, New York, NY 10021 USA

**Keywords:** Renal cell carcinoma, Pazopanib, Sunitinib

## Abstract

**Background:**

The international, phase 3 COMPARZ study demonstrated that pazopanib and sunitinib have comparable efficacy as first-line therapy in patients with advanced renal cell carcinoma, but that safety and quality-of-life profiles favor pazopanib. Our report analyzed pazopanib and sunitinib safety in Asian and non-Asian subpopulations.

**Methods:**

Patients were randomized 1:1 to receive pazopanib 800 mg once daily (continuous dosing) or sunitinib 50 mg once daily in 6-week cycles (4 weeks on, 2 weeks off).

**Results:**

Safety population was composed of 363 Asian patients and 703 non-Asian patients. Asian patients had similar duration of exposure to either drug compared with non-Asian patients, although Asian patients had a higher frequency of dose modifications. Overall, hematologic toxicities, cytopenias, increased AST/ALT, and palmar-plantar erythrodysesthesia (PPE) were more prevalent in Asian patients, whereas gastrointestinal toxicities were more prevalent in non-Asian patients. Among Asian patients, hematologic adverse events and most non-hematologic AEs were more common in sunitinib-treated versus pazopanib-treated patients. Among Asian patients, the most common grade 3/4 AEs with pazopanib were hypertension (grade 3, 22%) and alanine aminotransferase increased (grade 3, 12%; grade 4, 1%); the most common grade 3/4 AEs with sunitinib were thrombocytopenia/platelet count decreased (grade 3, 36%; grade 4, 10%), neutropenia/neutrophil count decreased (grade 3, 24%; grade 4, 3%) hypertension (grade 3, 20%), and PPE (grade 3, 15%).

**Conclusions:**

A distinct pattern and severity of adverse events was observed in Asians when compared with non-Asians with both pazopanib and sunitinib. However, the two drugs were well tolerated in both subpopulations.

**Trial registration:**

ClinicalTrials.gov, NCT00720941, Registered July 22, 2008

ClinicalTrials.gov, NCT01147822, Registered June 22, 2010

**Electronic supplementary material:**

The online version of this article (10.1186/s13045-018-0617-1) contains supplementary material, which is available to authorized users.

## Background

Orally administered tyrosine kinase inhibitors targeting angiogenesis are the standard-of-care for first-line systemic options for treatment-naive patients with advanced, predominantly clear-cell, renal cell carcinoma (RCC). Pazopanib and sunitinib are the most commonly used first-line agents in patients with advanced clear-cell RCC and a favorable or intermediate prognosis (per Memorial Sloan Kettering Cancer Center criteria) [1, 2].

Pazopanib and sunitinib exhibit differences in target kinase selectivity and affinity [3], approved dosing schedules [4–7], and the pharmacokinetics of absorption and clearance. A phase 3 study, COMPARZ, conducted to evaluate the relative efficacy and safety profiles of first-line pazopanib and sunitinib in patients with advanced or metastatic RCC, showed that the efficacy of these drugs is comparable, but that there were significant differences in safety profiles and patient quality-of-life [8, 9].

It is widely recognized that there are ethnic differences between Asian and non-Asian patients in physical attributes such as body surface area and body mass index, diet, cultural habits, and use of traditional medicines. Additionally, these ethnic groups differ in relative prevalence of functional polymorphisms in cytochromes and other genes [10–12] involved in drug absorption and metabolism. These factors potentially influence pharmacokinetic profiles and overall exposure and may result in differences in the overall safety profile of a drug in these populations. Accordingly, the current analysis of COMPARZ evaluated the relative safety profile of pazopanib and sunitinib in Asian and non-Asian patients.

## Methods

Detailed eligibility criteria, study design, efficacy endpoints, and statistical methods of the COMPARZ trial have been reported previously [8].

### Patients

Eligible patients were ≥ 18 years of age, with a diagnosis of locally advanced (not amenable to curative surgery or radiation therapy) or metastatic (equivalent to stage IV RCC according to American Joint Committee on Cancer staging) clear-cell RCC, who had not previously received systemic treatment for advanced or metastatic RCC. All patients had measurable disease per Response Evaluation Criteria In Solid Tumors (RECIST 1.0) [13], Karnofsky performance status (KPS) ≥ 70, and adequate hematologic, renal, and hepatic function.

The study was approved by the local institutional review boards. Patients provided written informed consent before any study-related procedures were performed. The study was conducted in accordance with Good Clinical Practice guidelines and the Declaration of Helsinki.

### Study design

The study was a randomized, open-label, parallel-group, phase 3 trial of pazopanib versus sunitinib in patients with advanced or metastatic RCC. The conduct of the trial, NCT00720941, is shown in Additional file 1 [8]. The first patient first visit (FPFV) in this trial was August 14, 2008. A substudy of similar design, NCT01147822, was conducted exclusively in Asian patients (Additional file 1) [8]. The FPFV in this substudy was May 19, 2010. The current analysis was based on pooled data from both studies.

Patients were stratified by KPS score (70–80 vs 90–100), baseline lactate dehydrogenase level (> 1.5 vs ≤ 1.5 × upper limit of normal), and previous nephrectomy (yes vs no). Patients were centrally randomized 1:1 to receive pazopanib 800 mg once daily (continuous dosing) or sunitinib 50 mg once daily in 6-week cycles (4 weeks on, 2 weeks off). Patients received treatment until disease progression per investigator (RECIST 1.0), death, unacceptable toxicity, or consent withdrawal for any reason.

### Endpoints and assessments

Safety assessments included physical examination, vital signs, hematology, clinical chemistry, coagulation tests, urine protein creatinine ratio, thyroid function tests, electrocardiogram, echocardiogram, and KPS scores at screening/baseline and periodically until discontinuation of study treatment. Clinical assessments for safety were evaluated in 6-week cycles based on the following schedule: on days 1, 14, 28, and 42 of cycle 1; on days 28 and 42 of cycles 2 through 9; and on day 42 of subsequent cycles. Prior to a protocol amendment, some safety assessments were performed on days 28 and 42 from cycle 10 until treatment discontinuation (Additional file 1 [8]). Adverse events (AEs) were graded according to National Cancer Institute Common Terminology Criteria for Adverse Events (CTCAE) version 3.0 [14].

### Statistical methods and analysis

Randomization to COMPARZ was not stratified according to Asian ethnicity. The Asian safety population was composed of all randomized patients who enrolled in Asian countries including China, Korea, Taiwan, and Japan, and who received at least one dose of study drug. The non-Asian safety population was composed of White randomized patients who identified as non-Asian and who received at least one dose of study drug. Patients of Asian ethnicity enrolled outside of Asia (*n* = 15) were not included in either of the analysis populations. A total of 19 patients who identified as African American/African heritage, American Indian, Alaska Native, and Alaska Native plus White also were excluded. Safety data were summarized using descriptive statistics.

## Results

### Patients

Between August 2008 and September 2011, 367 patients were enrolled from Asia and 707 non-Asian patients were enrolled from other regions. Although overall baseline demographics and disease characteristics were well balanced between treatment groups, there were small differences in time since initial diagnosis, KPS, number of organs involved, and number of metastatic sites (Table 1). Patient characteristics were balanced across the treatment arms (Table 2). The longer median duration of follow-up in non-Asian patients (pazopanib arm, 23.7 months; sunitinib arm, 22.9 months) than in Asian patients (pazopanib arm, 14.3 months; sunitinib arm, 14.1 months) may account in part for differences in disposition between studies NCT00720941 and NCT01147822.

**Table 1:** Summary of baseline demographic and disease characteristics (ITT population)

	Asian patients		Non-Asian patients	
	Pazopanib (*n* = 188)	Sunitinib (*n* = 179)	Pazopanib (*n* = 349)	Sunitinib (*n* = 358)
Age, median years (range)	59.0 (18–81)	58.0 (23–82)	62.0 (35–88)	63.0 (33–86)
Male sex, *n* (%)	137 (73)	137 (77)	247 (71)	265 (74)
Race, *n* (%)				
Asian—Central/South Asian heritage	2 (1)	1 (< 1)	0	0
Asian—East Asian heritage	154 (82)	144 (80)	0	0
Asian—Japanese heritage	29 (15)	31 (17)	0	0
Asian—South East Asian heritage	3 (2)	3 (2)	0	0
Non-Asian—White/Caucasian/European heritage	0	0	346 (99)	356 (99)
Non-Asian—Arabic/North African heritage	0	0	2 (< 1)	2 (< 1)
Non-Asian—mixed race	0	0	1 (< 1)	0
Time since initial diagnosis, median days (range)	93.5 (1–6039)	145.5 (2–6239)	287.0 (10–9117)	252.0 (9–7922)
Histology, *n* (%)				
Clear cell	183 (97)	165 (92)	322 (92)	331 (92)
Predominantly clear cell	5 (3)	10 (6)	20 (6)	20 (6)
Other	0	4 (2)	7 (2)	6 (2)
Missing	0	0	0	1 (< 1)
Karnofsky performance scale, *n* (%)				
70 or 80	37 (20)	20 (11)	96 (28)	103 (29)
90 or 100	151 (80)	159 (89)	253 (72)	255 (71)
Prior nephrectomy, *n* (%)	152 (81)	152 (85)	292 (84)	300 (84)
Baseline levels of LDH, *n* (%)				
> 1.5 × ULN	10 (5)	6 (3)	28 (8)	22 (6)
≤ 1.5 × ULN	178 (95)	173 (97)	321 (92)	336 (94)
Number of organs involved, *n* (%)				
1	53 (28)	50 (28)	61 (17)	54 (15)
2	71 (38)	67 (37)	127 (36)	131 (37)
≥ 3	63 (34)	62 (35)	161 (46)	173 (48)
Missing	1 (< 1)	0	0	0
Location of disease at baseline, *n* (%)				
Lung	146 (78)	143 (80)	262 (75)	270 (75)
Lymph nodes	62 (33)	68 (38)	153 (44)	172 (48)
Kidney	57 (30)	43 (24)	101 (29)	105 (29)
Bone	36 (19)	33 (18)	65 (19)	49 (14)
Other	27 (14)	27 (15)	45 (13)	50 (14)
Liver	24 (13)	30 (17)	60 (17)	78 (22)
MSKCC risk category, *n* (%)				
Favorable risk	47 (25)	55 (31)	97 (28)	90 (25)
Intermediate risk	119 (63)	112 (63)	192 (55)	209 (58)
Poor risk	18 (10)	9 (5)	48 (14)	41 (11)
Unknown	4 (2)	3 (2)	12 (3)	18 (5)
Heng risk category, *n* (%)				
Favorable risk	43 (23)	50 (28)	92 (26)	82 (23)
Intermediate risk	112 (60)	98 (55)	177 (51)	203 (57)
Poor risk	30 (16)	27 (15)	74 (21)	63 (18)
Unknown	3 (2)	4 (2)	6 (2)	10 (3)
Number of metastatic sites, *n* (%)				
0	1 (< 1)	3 (2)	2 (< 1)	2 (< 1)
1	77 (41)	57 (32)	91 (26)	77 (22)
2	58 (31)	72 (40)	130 (37)	146 (41)
≥ 3	51 (27)	47 (26)	126 (36)	133 (37)
Missing	1 (< 1)	0	0	0

*ITT* intent-to-treat, *LDH* lactate dehydrogenase, *MSKCC* Memorial Sloan Kettering Cancer Center, *ULN* upper limit of normal

**Table 2:** Patient disposition

	Asian patients		Non-Asian patients	
	Pazopanib	Sunitinib	Pazopanib	Sunitinib
Study discontinuation, *n* (%)	146 (78)	139 (79)	320 (92)	331 (93)
Died, *n* (%)	66 (35)	59 (33)	171 (49)	190 (53)

At data cutoff (May 21, 2012)

### Safety

#### Treatment exposure

The median time on study treatment for Asian patients was 8.4 months in the pazopanib arm and 8.0 months in the sunitinib arm and was numerically slightly higher versus non-Asian patients (7.2 months for pazopanib, 6.2 months for sunitinib). The relative mean dose of drug administered to Asian patients was similar in both arms at ~ 80% of planned dose (pazopanib, 634.1 mg; sunitinib, 40.1 mg) and was comparable with non-Asian patients (pazopanib, 686.6 mg; sunitinib, 41.8 mg).

#### Dose modifications and discontinuations

Similar proportions of Asian patients in the pazopanib and sunitinib groups had dose reductions (54 vs 59%) and interruptions (65 vs 64%). Non-Asian patients in the pazopanib and sunitinib groups had a slightly lower rate of dose reductions (40 vs 45%) and interruptions (57 vs 61%). The most common reasons for discontinuation of study treatment were disease progression and death (49 and 46% for pazopanib and sunitinib, respectively, in Asian patients; 53 and 60% for pazopanib and sunitinib, respectively, in non-Asian patients) and AEs (18 and 19% for pazopanib and sunitinib, respectively, in Asian patients; 27 and 19% for pazopanib and sunitinib, respectively, in non-Asian patients).

Among Asian patients, the predominant AE leading to treatment discontinuation of pazopanib was abnormal hepatic function tests (alanine aminotransferase [ALT] increased, 6%; hepatic function abnormal, 3%; aspartate aminotransferase [AST] increased, 2%). The most common AE leading to discontinuation of sunitinib was hematologic toxicity (thrombocytopenia/platelet count decreased, 12%; neutropenia/neutrophil count decreased, 3%; anemia/hemoglobin decreased, 2%).

#### Common adverse events

Among Asian patients, the most common treatment-emergent AEs (> 40% in either treatment arm) were hypertension, diarrhea, palmar-plantar erythrodysesthesia syndrome (PPE), fatigue, thrombocytopenia/platelet count decreased, neutropenia/neutrophil count decreased, leukopenia/WBC count decreased, anemia/hemoglobin decreased, ALT increased, AST increased, and decreased appetite. Adverse events occurring in ≥ 10% of patients in either treatment arm are presented in Table 3. Most AEs were grade 1 or 2. The proportion of patients who experienced maximum grade 3/4 AEs on the study was similar in both treatment arms (78% with pazopanib, 75% with sunitinib). In the Asian patients, the most common grade 3 AEs among pazopanib-treated patients were hypertension (22%), ALT increased (12%), and PPE (10%). The most common grade 3 AEs among sunitinib-treated Asian patients were thrombocytopenia/platelet count decreased (36%), neutropenia/neutrophil count decreased (24%), hypertension (20%), PPE (15%), and fatigue (11%); 10% experienced grade 4 thrombocytopenia/platelet count decreased. In the non-Asian patients, the most common grade 3 AEs among pazopanib-treated patients were hypertension (11%), fatigue (11%), and diarrhea (10%). The most common grade 3 AEs among sunitinib-treated non-Asian patients were hypertension (12%) and fatigue (20%).

**Table 3:** Summary of on-therapy treatment-emergent adverse events occurring in ≥10% of patients in either treatment arm (Safety population)

	Asian patients, n (%)						Non-Asian patients, n (%)					
	Pazopanib (*n* = 186)			Sunitinib (*n* = 177)			Pazopanib (*n* = 348)			Sunitinib (*n* = 355)		
Preferred Term	Any grade	Grade 3	Grade 4	Any grade	Grade 3	Grade 4	Any grade	Grade 3	Grade 4	Any grade	Grade 3	Grade 4
Neutropenia	44 (24)	11 (6)	1 (< 1)	77 (44)	25 (14)	5 (3)	15 (4)	4 (1)	1 (< 1)	68 (19)	26 (7)	1 (< 1)
Neutrophil count decreased	27 (15)	2 (1)	2 (1)	47 (27)	17 (10)	0	1 (< 1)	0	0	14 (4)	7 (2)	0
Neutropenia/neutrophil count decreased^a^	71 (38)	13 (7)	3 (2)	124 (70)	42 (24)	5 (3)	16 (5)	4	1	82 (23)	33 (9)	1
Leukopenia	40 (22)	5 (3)	0	59 (33)	10 (6)	0	11 (3)	0	0	38 (11)	4 (1)	0
WBC count decreased	24 (13)	1 (< 1)	0	52 (29)	4 (2)	0	4 (1)	0	0	19 (5)	4 (1)	0
Leukopenia/ WBC count decreased^a^	64 (34)	6 (3)	0	111 (63)	14 (8)	0	15 (4)	0	0	57 (16)	8	0
Platelet count decreased	30 (16)	5 (3)	0	62 (35)	25 (14)	5 (3)	5 (1)	0	0	38 (11)	7 (2)	1 (< 1)
Thrombocytopenia	31 (17)	8 (4)	2 (1)	81 (46)	39 (22)	12 (7)	24 (7)	0	1 (< 1)	100 (28)	23 (6)	7 (2)
Thrombocytopenia/platelet count decreased^a^	61 (33)	13 (7)	2 (1)	143 (81)	64 (36)	17 (10)	29 (8)	0	1	138 (39)	30	8
Hemoglobin decreased	21 (11)	0	0	48 (27)	8 (5)	1 (< 1)	12 (3)	4 (1)	0	21 (6)	4 (1)	0
Anemia	13 (7)	1 (< 1)	3 (2)	51 (29)	10 (6)	3 (2)	24 (7)	2 (< 1)	3 (< 1)	50 (14)	11 (3)	3 (< 1)
Anemia/hemoglobin decreased^a^	34 (18)	1 (< 1)	3 (2)	99 (56)	18 (10)	4	36 (10)	6	3	71 (20)	15	3

^a^Related AEs were pooled

Among Asian patients, AEs notably occurring more frequently with pazopanib compared with sunitinib were non-hematologic: hair color changes (36 vs 8%), increased gamma-glutamyltransferase (GGT) (10 vs 3%), alopecia (12 vs 6%), increased ALT (44 vs 32%), and increased AST (42 vs 33%). The frequency of several hematologic AEs was higher (any-grade difference of ≥ 20%) among sunitinib-treated patients compared with pazopanib-treated patients, and included leukopenia/WBC count decreased (63 vs 34%), thrombocytopenia/platelet count decreased (46 vs 17%), anemia/hemoglobin decreased (56 vs 18%), and neutropenia/neutrophil count decreased (70 vs 38%). Non-hematologic AEs occurring notably more frequently among Asian sunitinib-treated patients included stomatitis (30 vs 13%), PPE (64 vs 50%), hypothyroidism (28 vs 16%), increased blood creatinine (27 vs 17%), increased blood lactate dehydrogenase (24 vs 14%), constipation (18 vs 8%), blood thyroid-stimulating hormone increased (18 vs 9%), and yellow skin (24 vs 2%).

Among pazopanib-treated patients, Asians had higher incidences of several AEs compared with non-Asian patients, including any grade of PPE (50 vs 19%), AST increased (42 vs 19%), proteinuria (32 vs 10%), neutropenia/neutrophil count decreased (38 vs 5%), leukopenia/WBC count decreased (34 vs 4%), thrombocytopenia/platelet count decreased (33 vs 8%), ALT increased (44 vs 25%), bilirubin increased (19 vs 5%), and grade 3 hypertension (22 vs 11%). Conversely, Asian pazopanib-treated patients experienced lower incidences of AEs such as nausea (24 vs 56%), dysgeusia (13 vs 32%), fatigue (42 vs 61%), dyspnea (4 vs 19%), headache (15 vs 27%), and constipation (8 vs 21%). These differences in incidences of the above AEs in Asian versus non-Asian patients were also observed in sunitinib-treated patients.

#### Treatment-related adverse events

Among Asian patients, the most common drug-related AEs (> 40% in either treatment arm) were hypertension, diarrhea, PPE, ALT increased, fatigue, neutropenia/neutrophil count decreased, thrombocytopenia/platelet count decreased, leukopenia/WBC count decreased, and anemia/hemoglobin decreased. Drug-related AEs occurring in ≥ 20% of patients in either treatment arm are presented in Table 4. Among pazopanib-treated patients, Asians had higher incidences of several AEs compared with non-Asians, including hypertension (54 vs 37%), PPE (48 vs 19%), ALT increased (42 vs 23%), AST increased (38 vs 18%), proteinuria (31 vs 8%), neutropenia/neutrophil count decreased (36 vs 5%), thrombocytopenia/platelet count decreased (31 vs 8%), anemia/hemoglobin decreased (17 vs 6%), and leukopenia/WBC count decreased (32 vs 4%). Conversely, pazopanib-treated Asian patients experienced lower incidences of diarrhea (49 vs 63%), fatigue (40 vs 55%), nausea (22 vs 51%), and dysgeusia (13 vs 31%). Among sunitinib-treated patients, Asians compared with non-Asians had higher incidences of hypertension (51 vs 30%), proteinuria (29 vs 5%), neutropenia/neutrophil count decreased (68 vs 21%), leukopenia/WBC count decreased (62 vs 15%), thrombocytopenia/platelet count decreased (81 vs 35%), anemia/hemoglobin decreased (50 vs 14%), blood creatinine increased (24 vs 5%), hypothyroidism (28 vs 17%), blood lactate dehydrogenase increased (23 vs 1%), and yellow skin (24 vs 9%).

**Table 4:** Summary of on-therapy drug-related adverse events (any grade) occurring in ≥ 20% of patients in either treatment arm (Safety population)

	Asian patients, *n* (%)		Non-Asian patients, *n* (%)	
Preferred term	Pazopanib (*n* = 186)	Sunitinib (*n* = 177)	Pazopanib (*n* = 348)	Sunitinib (*n* = 355)
Neutropenia	41 (22)	74 (42)	15 (4)	62 (17)
Neutrophil count decreased	26 (14)	46 (26)	1 (< 1)	13 (4)
Neutropenia/neutrophil count decreased^a^	67 (36)	120 (68)	16 (5)	75 (21)
Leukopenia	37 (20)	58 (33)	9 (3)	38 (11)
WBC count decreased	23 (12)	51 (29)	3 (< 1)	16 (5)
Leukopenia/WBC count decreased^a^	60 (32)	109 (62)	12 (4)	54 (15)
Platelet count decreased	29 (16)	62 (35)	5 (1)	30 (8)
Thrombocytopenia	29 (16)	81 (46)	23 (7)	94 (26)
Thrombocytopenia/platelet count decreased^a^	58 (31)	143 (81)	28 (8)	124 (35)
Hemoglobin decreased	19 (10)	42 (24)	5 (1)	13 (4)
Anemia	12 (6)	46 (26)	15 (4)	38 (11)
Anemia/hemoglobin decreased^a^	31 (17)	88 (50)	20 (6)	51 (14)

^a^Related AEs were pooled

#### Serious adverse events

The frequency of serious adverse events (SAEs) related to study treatment was similar for Asian and non-Asian patients treated with pazopanib (24 vs 28%). The most common SAEs (> 2%) related to pazopanib treatment were ALT increased (5%) and hepatic function abnormal (3%) in the Asian subpopulation and ALT increased (7%) and AST increased (3%) in the non-Asian subpopulation.

The frequency of SAEs related to study treatment was higher in Asian versus non-Asian patients treated with sunitinib (33 vs 24%). The most common SAEs (> 2%) related to sunitinib treatment were thrombocytopenia/platelet count decreased (12%) and pyrexia (3%) in the Asian subpopulation and dehydration (3%) in the non-Asian subpopulation.

#### Fatal adverse events

Eleven patients died within 28 days of their last dose of study medication (3% in each treatment arm). Five deaths were attributed to the disease under study, five deaths were attributed to fatal SAEs (two in the pazopanib arm [cardiopulmonary failure and cerebral hemorrhage], three in the sunitinib arm [respiratory failure (2) and tumor hemorrhage]), and one death in the sunitinib arm was attributed to multiple causes (progressive disease, pancytopenia, and septic shock).

## Discussion

Duration of exposure to either pazopanib or sunitinib was similar among Asian patients and slightly higher than that of non-Asian patients. A slightly higher proportion of Asian patients required dose modifications (interruptions or reductions) compared with non-Asian patients. A slightly higher proportion of non-Asian patients on pazopanib discontinued the study drug due to AEs, compared with Asian patients on either arm and non-Asian patients on sunitinib. Overall, the AE profiles of both drugs were similar to those observed previously in patients with advanced RCC, and no new safety signals emerged.

As previously observed in the safety analysis of the overall intent-to-treat population of COMPARZ [8], the incidence of hematologic AEs among Asian patients was higher in those receiving sunitinib. These findings are consistent with higher incidence of fatigue in Asian patients receiving sunitinib. Moreover, compared with their non-Asian counterparts, Asian patients receiving sunitinib had higher incidence (≥ 20% difference in all grade) of PPE (64 vs 43%).

**Fig. 1:**
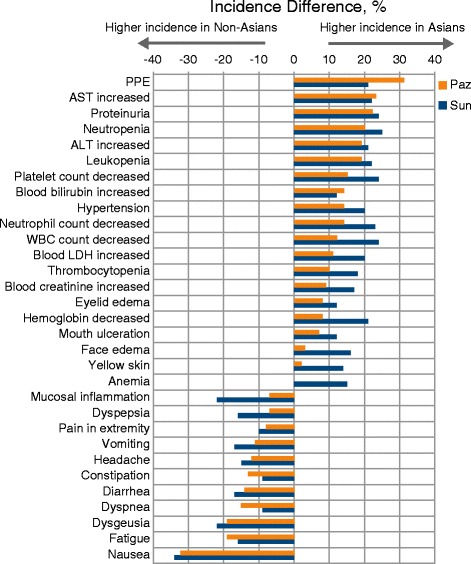
Adverse effects for which a frequency difference of ≥ 10% was observed between Asian and non-Asian populations in COMPARZ. ALT, alanine aminotransferase; AST, aspartate aminotransferase; LDH, lactate dehydrogenase; Paz, pazopanib; PPE, palmar-plantar erythrodysesthesia; Sun, sunitinib; WBC, white blood cells

In general, Asian patients experienced higher incidences of hypertension, hematologic toxicity, liver chemistry abnormalities, proteinuria, blood creatinine increased, and PPE with either drug compared with non-Asian patients. Conversely, non-Asian patients experienced higher incidences of gastrointestinal AEs, mucosal inflammation, headache, and dyspnea (Fig. 1).

Overall, the comparative AE profiles of pazopanib and sunitinib in the Asian population are consistent with the observation of increased hand, foot, and mouth soreness and fatigue among sunitinib-treated patients, as seen in the safety population of the parent study [8]. These data may reflect differences in ethnic tolerances for certain AEs. Differences in observed toxicity may be also due to ethnic differences in the ability to absorb and metabolize drugs [12, 15–17]. Further translational efforts are needed to provide insight into the contribution of these factors and to determine whether additional factors may be involved. For example, previous translational data have suggested the involvement of the hemochromatosis gene (*HFE*) [18] and *UGT1A1* [19] polymorphisms in hepatic enzyme elevation with pazopanib and vascular endothelial growth factor (*VEGF*) polymorphisms [20] with hypertension effects of sunitinib. A G allele variant of CYP1A1 (2455A>G) in patients with RCC has been associated with an increased risk of leukopenia [21]. The increased catalytic activity of this G allele variant and also CYP3A5 has been suggested to increase conversion of sunitinib to its metabolite, SU12662 [22, 23]; excessive accumulation of SU12662 has been associated with grade 3 thrombocytopenia and leukopenia [24]. CYP variants such as this are more prevalent in East Asian compared with European populations [25]. Similar investigations in the ethnic subpopulations of COMPARZ may provide useful guidance to enhance management of AEs associated with these drugs among specific ethnic groups, thereby improving patient quality of life.

This study has certain limitations. In this subgroup analysis of Asian patients, the Asian population was derived from selected countries in East Asia. Ethnic differences in susceptibility to the effects of chemotherapy are a well-recognized phenomenon, with both genetic and non-genetic factors influencing drug response [26, 27]. Therefore, generalization of the findings from this study across the entire Asian population requires caution. One limitation of this study concerns the effect of sunitinib treatment regimen (standard 4/2 schedule: sunitinib 50 mg/day; 4 weeks on treatment, 2 weeks off) on sunitinib safety profile. In real-world practice, patients receiving sunitinib are often initiated on alternative reduced dosing regimens to mitigate toxicity.

Previous studies have demonstrated no apparent differences in the pharmacokinetic profiles of both sunitinib and pazopanib in Asian versus non-Asian populations [28, 29]. In consideration of the comparable pharmacodynamic effects of drug exposure and treatment duration in Asian and non-Asian populations enrolled in COMPARZ, it is reasonable to conclude that therapeutic exposure to both drugs was achieved in both populations. Consistent with the parent study, the safety profile of pazopanib is more favorable than that of sunitinib in the context of symptomatic hematologic and nonhematologic toxicity. Adverse events associated with tolerability, such as hand-foot syndrome, stomatitis, and fatigue, were more common with sunitinib and have been reported to be of particular concern to patients with RCC [30, 31].

## Conclusion

In this subgroup analysis of the COMPARZ study, differential safety profiles of pazopanib and sunitinib in both Asian and non-Asian subpopulations were consistent with findings from overall population analysis. Although differences were observed in the incidences of AEs in Asians compared with non-Asians, pazopanib and sunitinib were well tolerated in both subpopulations.

## Additional file

Additional file 1: Conduct of NCT00720941 and NCT01147822 and timing of key study events. Protocol amendment 4 authorized inclusion of patients from NCT01147822 for safety and efficacy analyses. FPFV = first patient first visit. Data from Motzer RJ et al. [8]. Conduct of trials NCT00720941 and NCT01147822. (PDF 111 kb)

## Abbreviations

AE: Adverse event
ALT: Alanine aminotransferase
AST: Aspartate aminotransferase
CTCAE: Common Terminology Criteria for Adverse Events
FPFV: First patient first visit
GGT: Gamma-glutamyltransferase
KPS: Karnofsky performance status
PPE: Palmar-plantar erythrodysesthesia
RCC: Renal cell carcinoma
RECIST: Response Evaluation Criteria In Solid Tumors
SAE: Serious adverse events
