# Supplementary material for: Safety of pazopanib and sunitinib in treatment-naive patients with metastatic renal cell carcinoma: Asian versus non-Asian subgroup analysis of the COMPARZ trial
Source: J Hematol Oncol. 2018 May 22;11:69. doi: 10.1186/s13045-018-0617-1 (PMC5964681; doi:10.1186/s13045-018-0617-1)
Supplement: Supplementary file 1 — Conduct of NCT00720941 and NCT01147822 and timing of key study events. Protocol amendment 4 authorized inclusion of patients from NCT01147822 for safety and efficacy analyses. FPFV = first patient first visit. Data from Motzer RJ et al. [8]. Conduct of trials NCT00720941 and NCT01147822. (PDF 111 kb) [file 13045_2018_617_MOESM1_ESM.pdf]

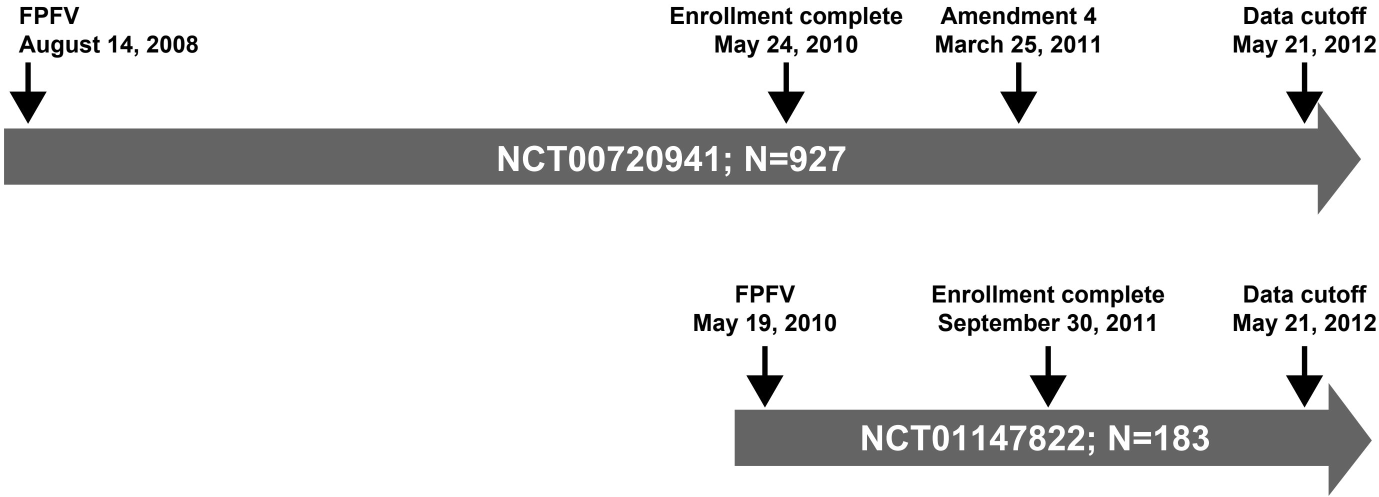
FPFV
August 14, 2008
Enrollment complete
May 24, 2010
Amendment 4
March 25, 2011
Data cutoff
May 21, 2012
NCT00720941; N=927
FPFV
May 19, 2010
Enrollment complete
September 30, 2011
Data cutoff
May 21, 2012
NCT01147822; N=183
